# Fractional Absorption of Active Absorbable Algal Calcium (AAACa) and Calcium Carbonate Measured by a Dual Stable-Isotope Method

**DOI:** 10.3390/nu2070752

**Published:** 2010-07-12

**Authors:** Kazuhiro Uenishi, Takuo Fujita, Hiromi Ishida, Yoshio Fujii, Mutsumi Ohue, Hiroshi Kaji, Midori Hirai, Mikio Kakumoto, Steven A. Abrams

**Affiliations:** 1 Laboratory of Physiological Nutrition, Kagawa Nutrition University. 3-9-21 Chiyoda, Sakado, Saitama 350-0288, Japan; 2 Katsuragi Hospital, 250-1 Makamicho, Kishiwada, Osaka 596-0842, Japan; Email: fujita@katsuragi-hosp.or.jp (T.F.); 3 Laboratory of Administrative Dietetics, Kagawa Nutrition University. 3-9-21 Chiyoda, Sakado, Saitama 350-0288, Japan; Email: ishida@eiyo.ac.jp; 4 Fujii Clinic, 4-18-1 Tsutsujigaoka, Tarumi-ku, Kobe, Hyogo 650-0853, Japan; Email: fujii-naika-clinic@crocus.ocn.ne.jp; 5 Division of Diabetes, Metabolism and Endocrinology, Department of Internal Medicine, Kobe University Graduate School of Medicine, 7-5-1 Kusunokichou, Chuo-ku, Kobe, Hyogo 650-0017, Japan; Email: hiroshik@med.kobe-u.ac.jp; 6 Department of Hospital Pharmacy and Pharmacodynamics, Kobe University Graduate School of Medicine, 7-5-1 Kusunokichou, Chuo-ku, Kobe, Hyogo 650-0017, Japan; Email: midorih@med.kobe-u.ac.jp (M.K.); 7 Department of Agriculture, Agricultural Research Service, Children’s Nutrition Research Center, Department of Pediatrics, Baylor College of Medicine and Texas Children’s Hospital, Houston, Texas 77030, USA; Email: sabrams@bcm.tmc.edu

**Keywords:** active absorbable algal calcium (AAACa), calcium carbonate, dual stable Ca isotope method, fractional absorption (FA), parathyroid hormone (PTH)

## Abstract

With the use of stable isotopes, this study aimed to compare the bioavailability of active absorbable algal calcium (AAACa), obtained from oyster shell powder heated to a high temperature, with an additional heated seaweed component (Heated Algal Ingredient, HAI), with that of calcium carbonate. In 10 postmenopausal women volunteers aged 59 to 77 years (mean ± S.D., 67 ± 5.3), the fractional calcium absorption of AAACa and CaCO_3_ was measured by a dual stable isotope method. ^44^Ca-enriched CaCO_3_ and AAACa were administered in all subjects one month apart. After a fixed-menu breakfast and pre-test urine collection (Urine 0), ^42^Ca-enriched CaCl_2_ was intravenously injected, followed by oral administration of ^44^Ca-enriched CaCO_3_ without carrier 15 minutes later, and complete urine collection for the next 24 hours (Urine 24). The fractional calcium absorption was calculated as the ratio of Augmentation of ^44^Ca from Urine 0 to Urine 24/ augmentation of ^42^Ca from Urine 0 to Urine 24. Differences and changes of ^44^Ca and ^42^Ca were corrected by comparing each with ^43^Ca. Fractional absorption of AAACa (mean ± S.D., 23.1 ± 6.4), was distinctly and significantly higher than that of CaCO_3 _(14.7 ± 6.4; p = 0.0060 by paired t-test). The mean fractional absorption was approximately 1.57-times higher for AAACa than for CaCO_3_. The serum 25(OH) vitamin D level was low (mean ± S.D., 14.2 ± 4.95 ng/ml), as is common in this age group in Japan. Among the parameters of the bone and mineral metabolism measured, none displayed a significant correlation with the fractional absorption of CaCO_3_ and AAACa. Higher fractional absorption of AAACa compared with CaCO_3_ supports previous reports on the more beneficial effect of AAACa than CaCO_3_ for osteoporosis.

## 1. Introduction

Active absorbable algal calcium (AAACa) prepared from heated oyster shell and seaweed is a unique calcium supplement with high bioavailability, with a characteristic lamellar crystalline structure quite unlike that of calcium oxide and calcium carbonate (CaCO_3_) [1]. In the Katsuragi Calcium study, a prospective, randomized, double blind and placebo-controlled study compared the effect of AAACa on osteoporosis with that of CaCO_3 _in hospitalized women with a mean age of 80 years. It was found that AAACa alone increased spinal bone mineral density significantly over the level in subjects given a placebo, whereas CaCO_3 _did not [2,3]. Fracture occurrence over the two year test period from among 58 subjects was 0 of 5 in the AAACa Group, 2 of 7 in the CaCO_3_ Group and 3 of 5 in the Placebo Group, on evaluation of all X-rays available at the beginning and end of the test period. The AAACa Group exhibited a significantly lower rate of fracture occurrence than the placebo group, but the CaCO_3_ Group showed no significant difference from placebo group. Serum parathyroid hormone (PTH) was also suppressed more efficiently by AAACa than CaCO_3._

Despite all these indirect lines of evidence indicating a high bioavailability of AAACa, a direct absorption test by a dual isotope method has not been conducted to date. We have therefore attempted to measure the fractional absorption of AAACa by using the dual stable-isotope method [4,5] to compare it with CaCO_3 _in subjects in the age group most likely to need effective calcium supplementation to maintain their bone health: postmenopausal women.

## 2. Experimental Section

### 2.1. Subjects

Ten postmenopausal women between 59 and 77 years of age (mean ± SD, 67 ± 5.3 years) leading a normal healthy daily life without any known disease possibly affecting bone and mineral metabolism volunteered to participate as test subjects in the present study by providing written consent (Table 1). One subject, shown in parenthesis in Table 1 and Table 2, was dropped from analysis because of a measured fractional absorption (FA) value of 0% on giving CaCO_3_. The Institutional Review Board of the Fujii Medical Clinic approved the study.

**Table 1:** Background of the test subjects.

No.	Age	Years after menopause	Height (cm)	Weight (kg)	Systolic blood pressure (mmHg)	Diastolic blood pressure (mmHg)
1	68	19	154	54	138	80
2	72	23	147	50	142	62
3	65	15	157	63	148	70
4	65	13	148	43	125	70
(5) *	(59)	(9)	(153)	(60)	(133)	(88)
6	59	8	152	58	152	85
7	65	13	151	56	150	85
8	77	28	150	48	142	80
9	64	15	145	50	140	90
10	71	19	148	48	122	70
**Mean**	67	17	150	52	139	76
**SD**	5.3	6.0	3.7	6.1	10.4	9.3

* Case No. 5 was not included in the statistical analysis.

### 2.2. Background Data of the Test Subjects

In order to assess the metabolic background of the test subjects, serum Ca, P, albumin, creatinine, BUN, 25(OH)vitamin D, intact parathyroid hormone (PTH), bone specific alkaline phosphatase (BAP), urinary N-terminal type I collagen fragments (NTx) and urinary calcium/ creatinine ratio (UCa/ Cr) were measured prior to the test. The laboratory tests related to bone and calcium metabolism gave results approximately within the normal range, as shown in Table 2, except for one subject, who had a serum 25(OH) vitamin D level in the insufficiency range (7.6 ng/mL). This subject was without symptoms and signs of vitamin D insufficiency such as hypocalcemia, hypophosphatemia, high alkaline phosphatase, muscle weakness and bone pain.

**Table 2:** Parameters of mineral and bone metabolism of the test subjects.

No.	Serum Ca	Serum P	Serum albumin	Serum creatinine	BUN	25(OH) vitamin D	Intact PTH	BAP	Urine NTx/Cr	Urine Ca/Cr
	mg/dL	mg/dL	g/dL	mg/dL	mg/dL	ng/dL	pg/dL	U/L	nMBCE/mMCr	mg/mg
1	9.7	3.9	4.4	0.83	11.0	16.8	48	15.2	32.2	0.03
2	9.5	4.5	4.0	0.80	21.9	16.9	31	15.1	16.0	0.06
3	9.7	3.1	4.6	0.71	12.1	11.6	40	35.8	31.1	0.45
4	10.3	3.5	5.1	0.49	12.5	11.7	44	32.4	35.9	0.36
5 *	(9.5)	(3.4)	(4.5)	(0.74)	(14.2)	(21.8)	(61)	(21.6)	(29.0)	(0.08)
6	9.8	3.5	4.7	0.74	17.8	15.4	44	19.1	23.0	0.12
7	9.3	4.4	4.4	0.60	17.8	24.7	42	17.0	42.2	0.22
8	9.9	2.9	4.6	0.59	13.5	7.6	50	34.9	34.5	0.17
9	9.8	3.7	4.4	0.74	13.7	12.7	34	27.5	16.3	0.20
10	9.5	3.3	4.2	0.54	13.6	10.8	34	45.9	21.9	0.30
**Mean**	9.7	3.6	4.5	0.67	14.9	14.2	41	27.0	28.1	0.21
**SD**	0.29	0.55	0.31	0.119	3.53	4.95	6.59	11.02	9.20	0.138

Ca: calcium; P: phosphorus; BUN: Blood urea nitrogen; PTH: parathyroid hormone; BAP: Bone specific alkaline phosphatase; BCE: Bone collagen equivalent. * Case No. 5 was not included in the statistical analysis

### 2.3. Materials

The first part of the test was performed on March 9, 2009, using ^44^Ca-enriched CaCO_3_ for oral load and ^42^Ca in the form of CaCl_2,_ for intravenous injection (Table 3). On April 13, 2009, after one month, exactly the same procedure was repeated on the same test subjects, except for the use of ^44^Ca-enriched AAACa in the place of CaCO_3_ to ensure the stable isotope constituent of the body reached equilibrium. Intrinsic labeling is no doubt ideal, but it is impossible to label the shell of oysters abiding in the ocean, so an extrinsic labeling was adopted as the best substitute for it. The material for AAACa was obtained by heating oyster shell to 1,000 ^o^C, resulting mostly in CaO powder after losing much of the organic components. To 5,082 mg of this CaO powder, 450.4 mg CaO Ca fraction was added that consisted of 95.9 ± 0.3% ^44^Ca supplied by TRACE SCIENCES INTERNATIONAL (Ontario, Canada), and was thoroughly mixed in a melting pot. Aqueous solution of a small amount of algal component was pre-heated at a high temperature in a manner similar to the oyster shell to start a chemical reaction lasting for about 10 minutes. After sufficient stirring, it was divided into small portions for actual use and preserved in vacuum. The final product mostly consisted of Ca(OH)_2_.

CaCO_3_ labeled with ^44^Ca was also obtained from the same source (the Ca fraction consisting of 95.9 ± 0.3% ^44^Ca). To 781.6 mg of this material, 9,075 mg CaCO_3_ (Japanese Pharmacopeia) was added, thoroughly mixed, and divided into small proportions and stored. 

AAACa particle mean size was 5.8 microns; maximum size was 75 microns and CaCO_3_ particle size ranged from 10 to 20 microns. As these values are based on different occasions of measurements they may not be directly comparable, but appears to lie over a similar range. If anything, a larger size is compatible with slower absorption. 

For two subjects, part of the first urine sample was lost; in these cases, both parts of the test were repeated on July 30 and August 27, and the data from the uneventfully performed second set of tests were used to replace those of the first set. 

The safety of the intravenous injection of CaCl_2_ was verified before the study by the absence of any signs of toxicity such as chills, fever, neuromuscular irritability, skin eruptions, disturbance of consciousness, *etc.*

**Table 3:** Amount of isotope Ca (mg) per subject in 1 study.

Isotope	Oral						IV
	CaX			CaY			Total
	Supplied	Added	Total	Supplied	Added	Total	
^42^Ca	0.01	1.79	1.80	0.00	1.79	1.79	3.192
^43^Ca	0.01	0.39	0.40	0.00	0.39	0.39	0.0037
^44^Ca	25.38	5.52	30.90	25.38	5.53	30.91	0.0334

The contents of Ca isotopes in the material used for the preparation of CaX and CaY on arrival from the supplier (Supplied), their contents in the material added to prepare samples for administration (Added) and the final total (Total) are indicated in Table 3.

A total of approximately 300 mg of Ca containing approximately 30 mg ^44^Ca isotope (25 + 5) was orally administered to each subject and about 3 mg ^42^Ca isotope was injected before the study and no symptoms and signs of toxicity were reported. 

### 2.4. Test Procedure

After taking a fixed menu breakfast consisting of fruit juice, toast, eggs and coffee, a pre-test urine sample was collected (Urine 0) and ^44^Ca-enriched CaCO_3_ was orally administered followed by the intravenous injection of ^42^Ca-enriched CaCl_2_ 15 minutes later. A complete collection of 24 h urine followed (Urine 24). After one month to ensure clearance of the enriched isotope, exactly the same procedure, except for the use of^ 44^Ca-enriched AAACa instead of ^44^Ca-enriched CaCO_3_, was repeated. 

#### 2.4.1. Measurement of the Stable Isotope

Sample preparation for isotope enrichment measurement was conducted according to the method of Patterson *et al*. [6]. By using the inductively coupled plasma mass spectrometry (ICP-MS, Agilent 7500 cs, Agilent Technologies, Inc., Tokyo), ^42^Ca, ^43^Ca, ^43^Ca and other measurable stable Ca isotopes were measured in both Urine 0 and Urine 24. Utilizing ^43^Ca as an internal standard of the stable Ca isotopes, the ratio of each stable Ca isotope to ^43^Ca was calculated. The increase of the ^42^Ca/^43^Ca and ^44^Ca/^43^Ca in Urine 24 above the pretest natural abundance level for each test subject over the corresponding value in Urine 0 was then obtained. By dividing the ratio of the actual amount of the enrichment of ^44^Ca by the corresponding amount of the enrichment of ^42^Ca from Urine 0 to Urine 24, the FA of the ^44^Ca-enriched material was obtained; for CaCO_3 _in the first part of the test and AAACa in the second part (Table 3).

### 2.5. Statistical Analysis

The Excel Statistical Package was used to compare the FA of CaCO_3_ and AAACa by paired t-test. A correlation matrix among the FA data, age and parameters of bone and mineral metabolism was constructed and evaluated by the Spearman method in view of the inclusion of variables with uncertain distribution. The p values < 0.05 were considered significant. 

## 3. Results and Discussion

As shown in Table 4, the mean Fractional absorption (FA) of AAACa, 23.1 ± 6.4%, was 1.57-times higher than the corresponding value of CaCO_3,_ 14.7 ± 6.4%, with a significant difference at p = 0.0060 determined using paired t-test.

**Table 4:** Fractional absorption (FA) of CaCO3 and AAACa by dual stable isotope method.

Subject	FA CaCO3	FA AAACa
1	7.5	21.1
2	20.0	29.7
3	21.9	34.7
4	19.6	20.4
(5) *	(0.0)	(18.8)
6	6.1	22.7
7	14.3	24.9
8	11.7	11.9
9	8.7	22.1
10	22.2	20.4
Mean	14.7	23.1
SD	6.4	6.4

Paired comparison between FA CaCO3 and FA AAACa p = 0.0060, t = 3.708 (paired t-Test) * Subject 5 was not included in the statistical analysis.

According to the evaluation by means of the correlation coefficient matrix (Spearman) (Table 5) among the parameters of bone and mineral metabolism summarized in Table 2, no significant correlation was found between the FA of either calcium carbonate or AAACa and each parameter. In the subject with the lowest serum 25(OH) vitamin D of 7.6 ng/mL, the FA of CaCO_3_ value was medium in the group, *i.e.*, 11.7%, fifth from the lowest, and the FA of AAACa, 11.9%, was the lowest in the group.

Until the advent of the dual isotope method, the true FA of calcium was extremely difficult to measure due to the complex behavior of calcium in living organisms, such as the rapid exchange through multiple Ca pools and various pathways of exit and reentrance [7,8]. Utilizing the presence of multiple stable isotopes in nature, the dual stable isotope method was developed to circumvent this complexity, and it is the only method of directly measuring the fractional intestinal Ca absorption.

Abrams and coworkers as well as other investigators [12,13,14,15,16,17,18,19,20,21,22,23] have used this method extensively to estimate calcium absorption, establishing it as the gold standard for calcium absorption. Since calcium absorption is influenced by age and the state of bone, as well as mineral metabolism, a correlation matrix was constructed and evaluated by Spearman’s method (Table 5). None of the metabolic parameters tested exhibited significant correlation with FA of the calcium compounds. Absence of significant correlation between FA of calcium compound and age was expected because of the narrow age range of this group.

The FA of Ca compounds obtained in this study of postmenopausal women, with a mean age of 66 years and with a tendency of low 25(OH) vitamin D, appears to be much lower than those observed in children and younger subjects: FA; 54.8-63.1% [21], 58.2-64.3% [22], and also younger postmenopausal women with mean age of 56: FA; 34.6-39.1% [23]. In healthy volunteers between 25 to 45 years much lower values, yet still higher than the results in the present study, were reported: FA; 26-31% [24]. The reduced FA in the current study subjects could also be due to reduced estrogen level after menopause. FA is, thus, markedly influenced by age. The age range of the test subjects was quite narrow in this group of subjects, unsuitable for the assessment of the age-FA correlation. Statistically, the tendency of age-FA correlation was non-significant.

**Table 5:** Spearman’s correlation matrix and correlation coefficients among fractional absorption (FA) and parameters of bone and mineral metabolism.

	FA CaCO3	FA AAACa	Age	SCa	SP	Salb	Cre	BUN	25D	PTH	BAP	UNTx	UCa/Cr
FA CaCO3	1.0000	0.1423	0.4238	−0.3882	−0.1925	−0.3405	−0.4854	−0.0335	−0.3167	−0.5630	−0.4833	−0.1333	0.6333
FA AAACa		1.0000	−0.3490	−0.5297	0.4328	−0.2137	0.5042	0.3445	0.5690	−0.5232	−0.3766	−0.2762	0.0418

SCa: serum calcium; SP: serum phosphate; Salb: serum albumin; Cre: creatinine; BUN: blood urea nitrogen; 25D: 25(OH)vitaminD; PTH: parathyroid hormone; BAP: bone specific alkaline phosphatase; UNtx: urine N-terminal type I collagen fragments; UCa/Cr: urinary Ca/creatinine ratio

Although these subjects are reasonably homogeneous and apparently free of any comorbidity, which could potentially influence the test results, the present study is limited by the small number of test subjects. Unlike similar studies conducted in this field in the past, post-menopausal women - who need calcium supplementation most because of high risk of osteoporosis - were asked to participate. A rather low intra-group variation was encouraging, and a clear-cut difference in FA between the two test materials may also add to the credibility of the conclusion.

It is possible that the difference in molecular weight and physicochemical properties of the ^44^Ca-enriched CaCO_3_ and AAACa, mostly consisting of Ca(OH)_2_ as the result of oxidation of CaCO_3_ obtained from oyster shell, cannot be completely ruled out. The similar molecular size and comparable particle size actually measured as 5.8 to 75 for AAACa and 10 to 20 for CaCO_3_ and this is a limitation of the result but should not have affected the primary outcome. In view of the similar molecular size and physicochemical properties between CaCO_3_ and AAACa, both much smaller than organic Ca salts, however, confounding effect exerted on the calculation of the absorptive rate is rather unlikely and the conclusion of difference in the absorption rate between the two compounds should be reasonably supported. 

## 4. Conclusions

This study aimed to compare the bioavailability of active absorbable algal calcium (AAACa), oyster shell powder heated to a high temperature, with an additional heated seaweed component (Heated Algal Ingredient, HAI), with that of calcium carbonate. The Fractional absorption of AAACa, (mean ± S.D.; 23.1 ± 6.4) was distinctly and significantly higher than that of CaCO_3_ (14.7 ± 6.4; p = 0.0060 by paired t-test). The mean was approximately 1.57-times higher for AAACa than CaCO_3_. Higher fractional absorption of AAACa compared with CaCO_3_ supports previous reports on the more beneficial effect of AAACa than CaCO_3_ on osteoporosis.
